# Assessment of infection prevention and control practices in healthcare facilities in the O.R. Tambo district municipality using the WHO infection prevention and control assessment framework

**DOI:** 10.3389/fpubh.2026.1772386

**Published:** 2026-06-05

**Authors:** Didi Monwabisi, Mojisola Clara Hosu, Yomelela Zibele Jason Mnqabashe, Mirabel Kah-Keh Nanjoh

**Affiliations:** 1 School of Public Health, Faculty of Medicine and Health Sciences, Walter Sisulu University, Mthatha, South Africa; 2 School of Pathology, Faculty of Medicine and Health Sciences, Walter Sisulu University, Mthatha, South Africa; 3 Biostatistics Analytic Training Services Unit, Walter Sisulu University, Mthatha, South Africa

**Keywords:** antimicrobial resistance, healthcare systems, healthcare-associated infections, infection prevention and control, O.R Tambo, patient safety, WHO IPCAF

## Abstract

**Background:**

Infection prevention and control (IPC) programs are essential for reducing healthcare-associated infections (HAIs), improving patient safety, and strengthening health systems. In low-resource settings, inadequate IPC capacity remains a major challenge, particularly at primary healthcare facilities that serve as the first point of contact in the health system. This study assessed IPC practices and capacity in healthcare facilities in the O.R. Tambo District Municipality using the World Health Organization (WHO) Infection Prevention and Control Assessment Framework (IPCAF).

**Method:**

A cross-sectional descriptive study was conducted at primary healthcare facilities in the O.R. Tambo District Municipality. Eighteen facilities were targeted for participation; 13 consented, yielding a response rate of 72.2%. Data were collected using the WHO IPCAF tool, which assesses eight core IPC components. Descriptive statistics summarized facility characteristics, IPCAF scores, and IPC capacity levels.

**Result:**

Most facilities [69.2% (95% CI: 42.3–88.6%, *n* = 9)] were classified as intermediate IPC level, while 15.4% (95% CI: 3.3–40.9%, *n* = 2) were inadequate, and 7.7% (95% CI: 0.8–30.7%, *n* = 1) each were categorized as basic and advanced. The overall median IPCAF score was 452.5 (IQR: 425.0–471.0), indicating intermediate IPC capacity. Higher median scores were observed for IPC programs, IPC guidelines, and the built environment, materials, and equipment domains, whereas HAI surveillance had the lowest median score. IPC education and training, as well as workload, staffing, and bed occupancy, scored below 50. All facilities had an IPC program in place; however, only 46.2% had clearly defined objectives and annual activity plans. IPC committees were present in 76.9% of facilities, and senior leadership participation was reported in 92.3%. Institutional budgetary support for IPC was available in only 15.4% of facilities.

**Conclusion:**

Healthcare facilities in the O.R. Tambo District Municipality predominantly had intermediate IPC capacity, with notable gaps in HAI surveillance, staffing, training, and institutional financial support. Strengthening these areas is essential to improve IPC capacity and enhance patient safety in resource-limited healthcare settings.

## 1 Introduction

Infection prevention and control (IPC) is fundamental to delivering safe healthcare, aiming to reduce healthcare-associated infections (HAIs) and limit the spread of antimicrobial resistance (1, 2). Evidence suggests that well-implemented IPC interventions can significantly reduce HAIs and antimicrobial resistance in healthcare settings (3). Globally, HAIs affect millions of patients each year, with a disproportionate burden in low- and middle-income countries (LMICs) due to gaps in infrastructure, resources, and adherence to IPC standards. Consequently, the risk of acquiring HAIs in LMICs is estimated to be up to 20 times higher than in high-income countries (HICs) (4–6). In response to the growing burden of HAIs, WHO developed the Infection Prevention and Control Assessment Framework (IPCAF) to support standardized assessment of IPC programs at the healthcare facility level. The tool evaluates eight core IPC components, including IPC programs, guidelines, training, surveillance, multimodal strategies, monitoring and auditing, workload and staffing, and the built environment (7). The WHO Global Report on IPC (2024) highlighted persistent deficiencies in facility-level IPC implementation, particularly in resource-constrained settings, where low-income countries (LICs) had notably limited implementation of IPC guidelines, training and education, monitoring, audit, feedback, and HAI surveillance. The report indicates that only 35.7% of LIC facilities met at least half of the requirements, and just 0.6% met all of them. In comparison, high-income countries (HICs) performed significantly better, with 98.8% meeting at least 50% of the requirements and 27.9% meeting all of them (8).

In South Africa, national IPC guidelines and policies are in place; however, evidence on how they translate into facility-level IPC capacity, particularly in rural and resource-constrained settings, is limited (9, 10). Existing reports often provide broad national or provincial insights but do not adequately capture facility-specific IPC capacity using standardized tools such as IPCAF. In the O.R Tambo District Municipality, a predominantly rural area with documented challenges in the healthcare system, there is a notable lack of empirical data on facility-level IPC capacity using the WHO IPCAF tool. This gap limits health authorities’ ability to identify specific weaknesses, prioritize interventions, and monitor progress in strengthening IPC systems.

Given the persistent challenge of HAIs and the need for targeted, evidence-based strategies, a structured facility-level assessment of IPC capacity is crucial. This data is vital for informing policy decisions, directing resource allocation, and improving patient safety outcomes in the district. Consequently, this study aims to assess IPC capacity in healthcare facilities within the O.R Tambo District Municipality using the WHO IPCAF tool to identify key strengths and gaps and to highlight priority areas for targeted IPC improvements.

## 2 Materials and methods

### 2.1 Study design, setting, population, and sampling

This cross-sectional, descriptive study was conducted at public health care facilities (HCFs) in the O.R. Tambo district municipality. The study focused on primary-level HCFs that serve as patients’ first point of contact with the health system. The study population comprised 11 community health centers (CHCs) that provide primary health care and facility-based services, including 24 h maternity care, emergency and casualty services, and short-stay wards; nine district hospitals that offer outpatient and inpatient clinical and allied health services; and two regional hospitals that provide specialist outpatient and inpatient services across multiple disciplines (11). These facilities totaled 22, and adherence to IPC practices was assessed using the WHO IPCAF tool (Table 1).

**Table 1:** Healthcare facility distribution by type and sub-district location in the O.R. Tambo district municipality.

Sub-district municipality; *N* = 5	Level 1 healthcare facilities		Level 2 healthcare facilities
	Community health centers; *N* = 11	District hospitals; *N* = 9	Regional hospitals; *N* = 2
King Sabata Dalindyebo	1. CHC1	1. DH1	1. RH1
	2. CHC2		
	3. CHC3		
	4. CHC4		
	5. CHC5		
Port St. Johns	6. CHC6	2. DH2	
	7. CHC7		
Nyandeni	8. CHC8	3. DH3	
		4. DH4	
Mhlontlo	9. CHC9	5. DH5	
	10. CHC10	6. DH6	
		7. DH7	
Ingquza Hill	11. CHC11	8. DH8	2. RH2
		9. DH9	

Although Primary Health Care Clinics (PHCs) are classified as primary-level healthcare facilities within South Africa’s four-tier referral system (11), they were excluded from this study because a dedicated WHO tool exists to assess IPC minimum requirements in outpatient and primary healthcare settings, including PHCs (12). Additionally, HCFs outside the O.R Tambo District Municipality were excluded.

A stratified random sampling technique was employed to select participating HCFs, with strata defined by level of care. Proportional allocation was performed using the probability-proportional-to-size formula: $n_{i}=(N_{i}/N)\;$ to determine the number of facilities selected from each stratum (Table 2). Facilities were stratified into CHCs, district hospitals, and regional hospitals, and simple random sampling was applied within each stratum to ensure unbiased selection. In cases of repeated non-response or refusal from selected facilities, replacement facilities were approached.

**Table 2:** Distribution of population size and proportional allocation across healthcare facility types.

Facility type	Population size; (*N_i_*)	Expected proportion to size; *n*_i_	Expected population; *n*_i_	Sample reached
CHCs	11	11/22 * 18 = 9	9	8
District hospitals	9	9/22 * 18 = 7.4	7	5
Regional hospitals	2	2/22 * 18 = 1.6	2	0
Total	22	18	18	13

### 2.2 Sample size calculation

**Figure 1:**
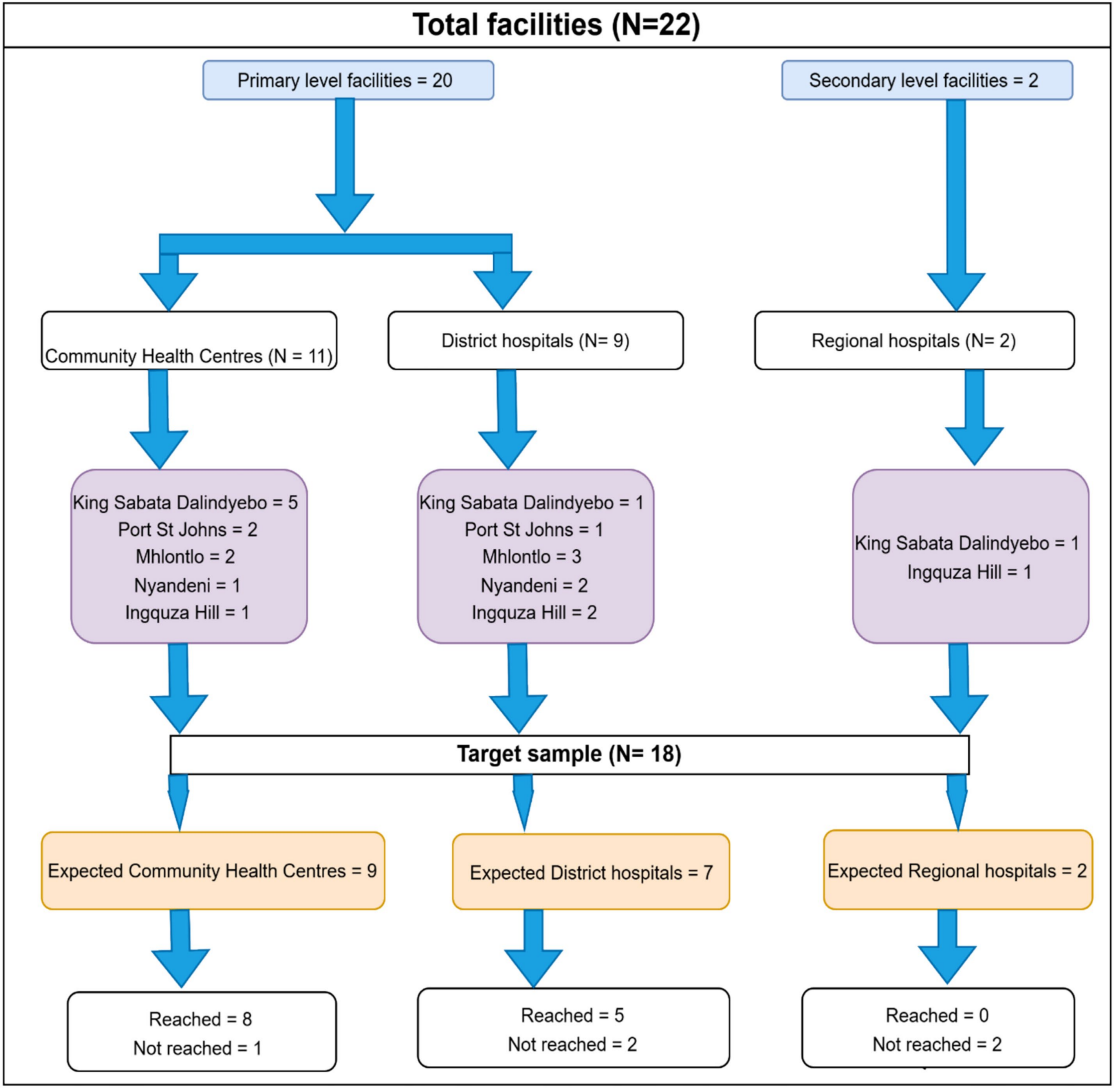
Sampling flowchart showing facility distribution, allocation, and recruitment.

The probability-proportional to size formula ( $n_{i}=(N_{i}/N)*n$ ) was used to determine the population size for each facility type. Sample size was estimated using EPIDAT based on the total of 22 CHCs, district, and regional hospitals in the O.R Tambo District Municipality. In the absence of comparable IPC prevalence data in South Africa, as the NDoH (9) reports only scores, a conservative 50% proportion was assumed. A 95% confidence level and 10% absolute precision were applied; the latter was appropriate for the small population and permitted random sampling rather than a full census, given that smaller margins require larger samples. The final sample size was 18 facilities (9 CHCs, 7 district hospitals, and 2 regional hospitals). However, because participation was voluntary, the final sample comprised 13 facilities, each with representatives who consented to participate in the study (Figure 1). Five eligible facilities (1 CHC, 2 district hospitals, and 2 regional hospitals) declined to participate despite repeated contact during the data collection period.

### 2.3 Data collection, management, and analysis

Data were collected from HCF managers or their representatives using the WHO pre-validated IPCAF tool (7). The tool was administered using an interviewer-guided approach to ensure completeness and consistency. Data collection was conducted by five trained sub-district quality assurance personnel, each representing a sub-district municipality, following standardized procedures. Clarifications were provided during data collection, as needed, to ensure contextual relevance without altering the tool’s structure, wording, or scoring system. Data collection took place from January 16, 2025, to March 15, 2025.

#### 2.3.1 Study instrument

The pre-validated WHO IPCAF tool was used to assess IPC practice levels, as recommended by WHO, in CHCs, district hospitals, and regional hospitals in the O.R Tambo district municipality. The tool is a structured, closed-format questionnaire with a scoring system comprising eight IPC system core components, including IPC program, IPC guidelines, IPC education, HAI surveillance, multimodal strategies, monitoring/audit of IPC practices and feedback, workload, staffing, and bed occupancy; and environments, materials, and equipment, and 81 measurable indicators (7). Each core component has indicators with associated scores. Each section generates a score between 0 and 100. The total score for each health facility is the sum of the scores for all core components. Based on the final score (ranging from 0 to 800), the facility’s IPC program implementation is categorized into four levels: inadequate (0–200), basic (201–400), intermediate (401–600), or advanced (601–800). An IPC level is assigned to an HCF based on its overall score.

#### 2.3.2 Data quality assurance

Data quality was ensured through standardized data collection procedures and prior training for data collectors on the IPCAF tool’s use and interpretation. Completed assessment forms were reviewed on-site for completeness and accuracy. During data entry, records were cross-checked to identify and resolve discrepancies. Data cleaning included checks for missing values, duplicate entries, and logical inconsistencies, with verification against the original records before analysis.

#### 2.3.3 Validity and reliability

The WHO IPCAF is a globally standardized and validated instrument with inherent content and construct validity. The tool has demonstrated excellent reliability, with internal consistency and inter-observer reliability (intra-class correlation coefficient) of 0.92 (95% CI: 0.89–0.94) (2). It was used in its original form without modification to preserve standardization and comparability. Although not formally psychometrically validated in South Africa, the IPCAF has been widely used in comparable low- and middle-income settings (13–16). Formal statistical reliability testing was not conducted in this study, given the instrument’s established validity and prior evidence of reliability.

#### 2.3.4 Ethical considerations

Ethical clearance was obtained from the Walter Sisulu University Health Sciences Research Ethics Committee (protocol code 233/2024; approval date 12 November 2024). Administrative approval was also obtained from the Eastern Cape Department of Health, the O.R Tambo District Department of Health, and management of the HCFs. Written informed consent was obtained from HCF managers or their designated representatives prior to data collection, confirming institutional participation. Participation was voluntary, and participants could withdraw at any time without penalty. To ensure confidentiality, no identifying information was collected. All data were anonymized and securely stored. Hard-copy questionnaires were kept in a locked cabinet, and electronic data were stored in password-protected files accessible only to the research team.

### 2.4 Statistical analysis

Data was entered into Microsoft Excel and analyzed using SPSS version 30 (IBM Corp., Armonk, NY, United States). All descriptive statistics for HCFs are presented as frequencies and percentages, including the facility’s location and level. The outcome variable was the IPCAF category achieved by each participating HCF, presented in tabular form. The secondary outcomes were the continuous IPCAF core component scores (CC1–CC8), each rated on a 0–100 scale.

Normality of continuous IPCAF scores was assessed using the Shapiro–Wilk test. Although the data were approximately normally distributed, nonparametric tests were used because of small, unequal group sizes and to ensure robust inference.

The median IPCAF total score and the median scores for the individual core components were computed to determine the overall level of IPC implementation. WHO-defined IPCAF scores were used to categorize facilities (inadequate, basic, intermediate, or advanced), and the proportions in each category were presented in tables and illustrated with bar graphs. The evaluation of the WHO IPC core components and indicators in HCF is presented in tables as frequencies and percentages.

Comparisons of overall IPCAF categories across facility types and locations were conducted using Fisher’s exact test (Fisher–Freeman–Halton approach with Monte Carlo simulation). Comparisons of continuous core component scores between two independent groups were performed using the Mann–Whitney *U* test, and comparisons across more than two groups were conducted using the Kruskal–Wallis test. A *p*-value of less than 0.05 was considered statistically significant for all inferential statistics.

## 3 Results

### 3.1 Characteristics of participating healthcare facilities and participants

#### 3.1.1 Characteristics of the participating healthcare facilities

Of the 18 HCFs targeted for participation, only 13 consented, yielding a response rate of 72.2%. The participating facilities comprised CHCs (61.5%) and district hospitals (38.5%), with no regional hospitals represented in the final sample. Facilities were distributed across five sub-districts, with the Mhlontlo sub-district contributing the largest share (38.5%) (Table 3).

**Table 3:** Characteristics of the participating healthcare facilities.

Variable	Category	*n* (%); *N* = 13
Facility type	CHC	8 (61.5)
	District hospital	5 (38.5)
	Total	13 (100.0)
Location	Port St. Johns	2 (15.4)
	Mhlontlo	5 (38.5)
	King Sabata Dalindyebo	4 (30.8)
	Nyandeni	1 (7.7)
	Ingquza Hill	1 (7.7)

#### 3.1.2 Characteristics of the participants

Participants were healthcare workers assigned IPC responsibilities, with IPC nurses comprising the majority (76.9%). Across facility types and localities, IPC nurses were represented in most settings, whereas all quality assurance personnel were in district hospitals (Table 4).

**Table 4:** Characteristics of the participating healthcare facilities by the professional role of participants.

Characteristic	Category	IPC nurse, *n* (%) *N* = 10	Quality assurance personnel, *n* (%) *N* = 3
Facility type	CHCs	8 (80.0)	0 (0.0)
	District hospitals	2 (20.0)	3 (100.0)
	Total	10 (76.9)	3 (23.1)
Local municipality	Port St. Johns	2 (20.0)	0 (0.0)
	Mhlontlo	4 (40.0)	1 (33.3)
	King Sabata Dalindyebo	3 (30.0)	1 (33.3)
	Nyandeni	0 (0.0)	1 (33.3)
	Ingquza Hill	1 (10.0)	0 (0.0)

### 3.2 Assessment of IPC core component scores across healthcare facilities in the OR Tambo District municipality

**Figure 2:**
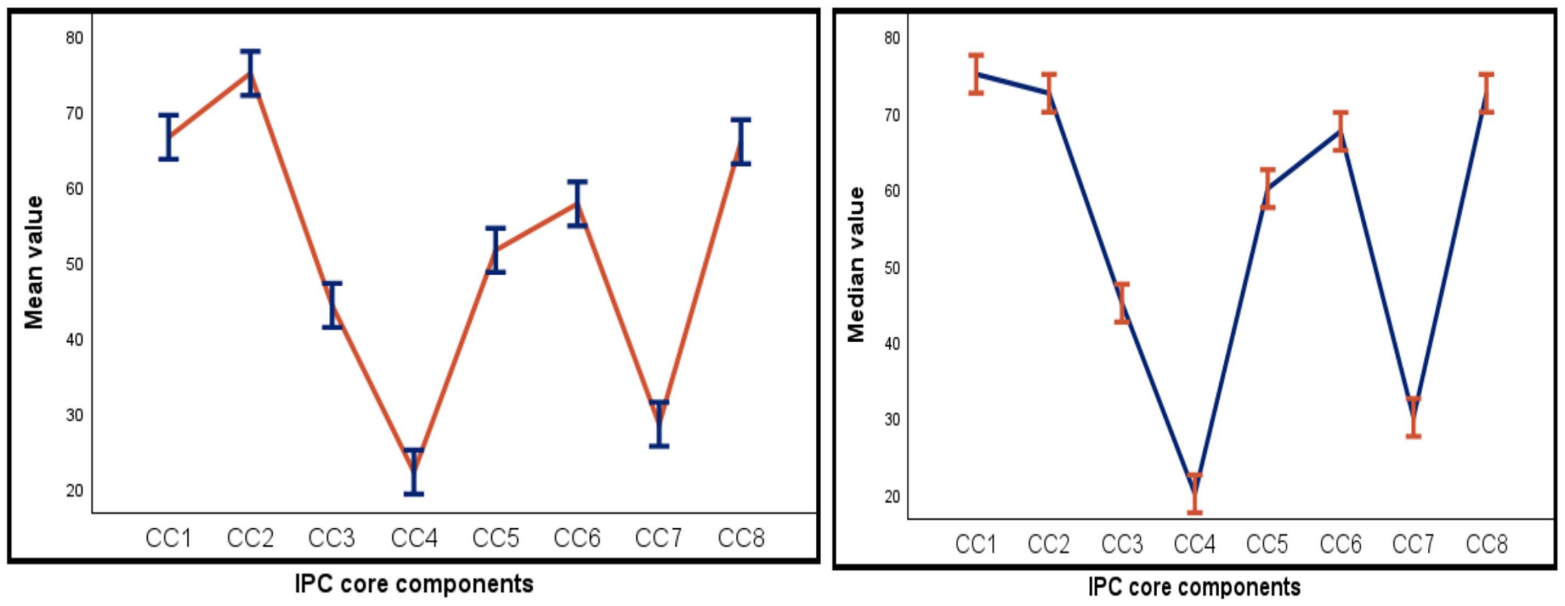
Mean and median IPC score stratified by core components.

Based on the core component score distribution, the highest median score (75.0) was recorded for IPC programs (CC1), followed by IPC guidelines (CC2) and built environment, materials, and equipment for IPC (CC8), both at 72.5. HAI (CC4) had the lowest score [median 20.0, IQR: 0.0–32.5] (Figure 2). The 13 hospitals had a median IPCAF score of 452.5, corresponding to an intermediate IPC level (Table 5).

**Table 5:** Median WHO IPCAF core component scores across HCFs in the OR Tambo district municipality.

WHO IPCAF core component (CC)	Median (IQR)
CC1: IPC program	75.0 (57.5–82.5)
CC2: IPC guidelines	72.5 (70.0–80.0)
CC3: IPC education and training	45.0 (45.0–60.0)
CC4: Healthcare-associated infection surveillance	20.0 (0.0–32.5)
CC5: Multimodal strategies for IPC implementation	60.0 (60.0–65.0)
CC6: Monitoring and audit of IPC practices and feedback	67.5 (47.5–75.0)
CC7: Workload, staffing, and bed occupancy	30.0 (20.0–30.0)
CC8: Built environment, materials, and equipment for IPC	72.5 (62.5–77.5)
Total IPCAF score	452.5 (425.0–471.0)

**Figure 3:**
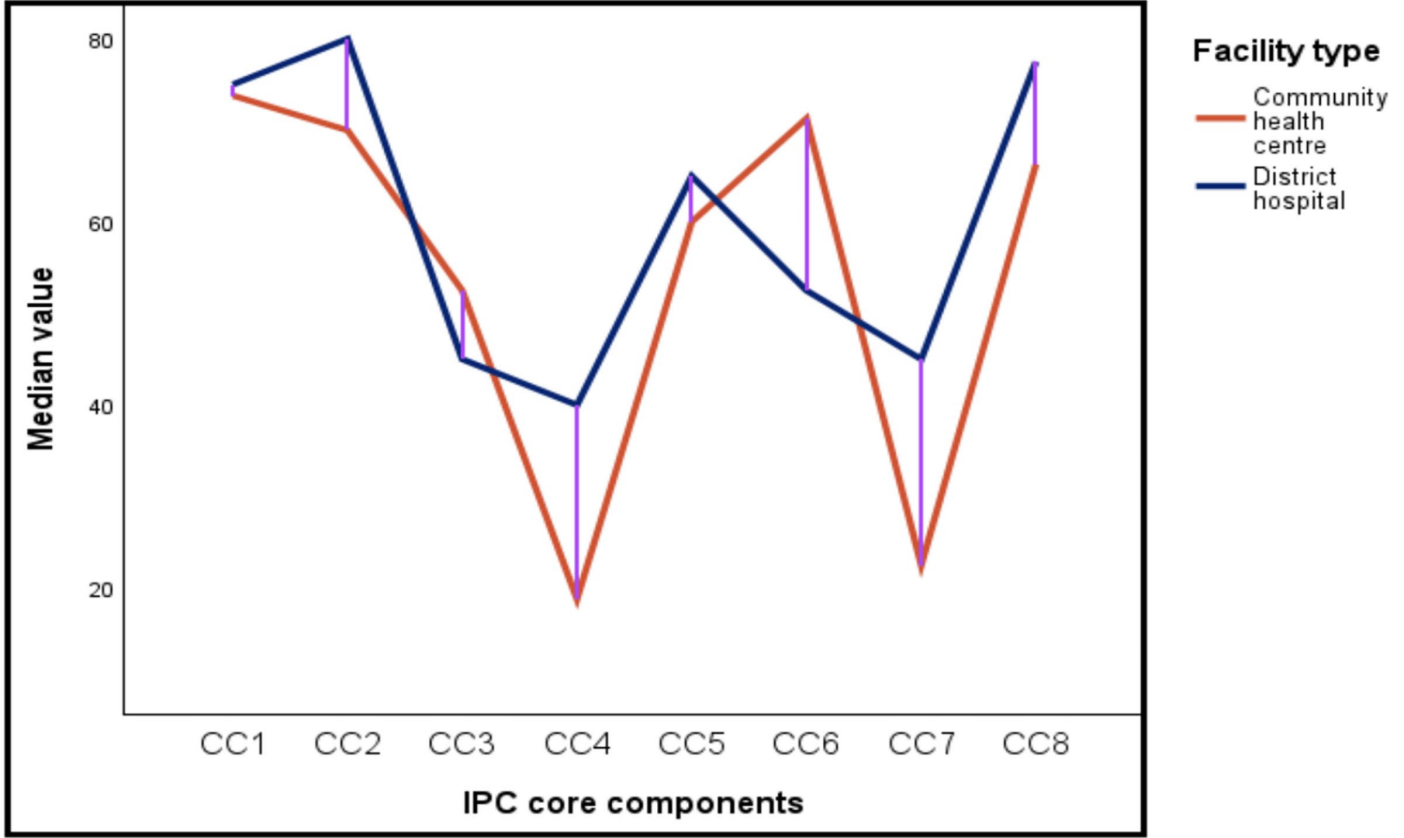
Median IPCAF scores stratified by core components and HCF type.

District hospitals outperformed CHCs across six IPCAF core components, with the largest statistically significant differences observed for workload, staffing, and bed occupancy (CC7) and for the built environment, materials, and equipment for IPC at the facility level (CC8) (Table 6). CHCs scored higher only in IPC education and training and in monitoring and auditing IPC practices (Figure 3). The overall IPCAF score for district hospitals was 471, compared with 452.5 for CHCs (Table 6).

**Table 6:** Comparison of the IPC scores between CHCs and the district hospital.

Core components	Facility type; median (interquartile range)		Mann–Whitney *U p*-value
	CHC; *N* = 8	District hospital; *N* = 5	
CC1: IPC program	73.8 (50.0–82.5)	75.0 (57.5–77.5)	1.000
CC2: IPC guidelines	70.0 (68.8–76.3)	80.0 (77.5–90.0)	0.171
CC3: IPC education and training	52.5 (25.0–60.0)	45.0 (45.0–45.0)	0.724
CC4: health care-associated infection (HAI) surveillance	18.8 (8.75–25.0)	40.0 (0.0–52.5)	0.435
CC5: multimodal strategies for implementation of IPC interventions	60.0 (47.5–62.5)	65.0 (60.0–65.0)	0.524
CC6: monitoring/audit of IPC practices and feedback	71.3 (53.8–75.0)	52.5 (47.5–57.5)	0.435
CC7: workload, staffing, and bed occupancy	22.50 (10.0–30.0)	45.0 (30.0–55.0)	**0.030**
CC8: built environment, materials, and equipment for IPC at the facility level	66.3 (48.8–72.5)	77.50 (75.0–78.5)	**0.045**
Total IPCAF score	452.5 (312.5–464.8)	471.0 (432.5–501.0)	0.284

**Figure 4:**
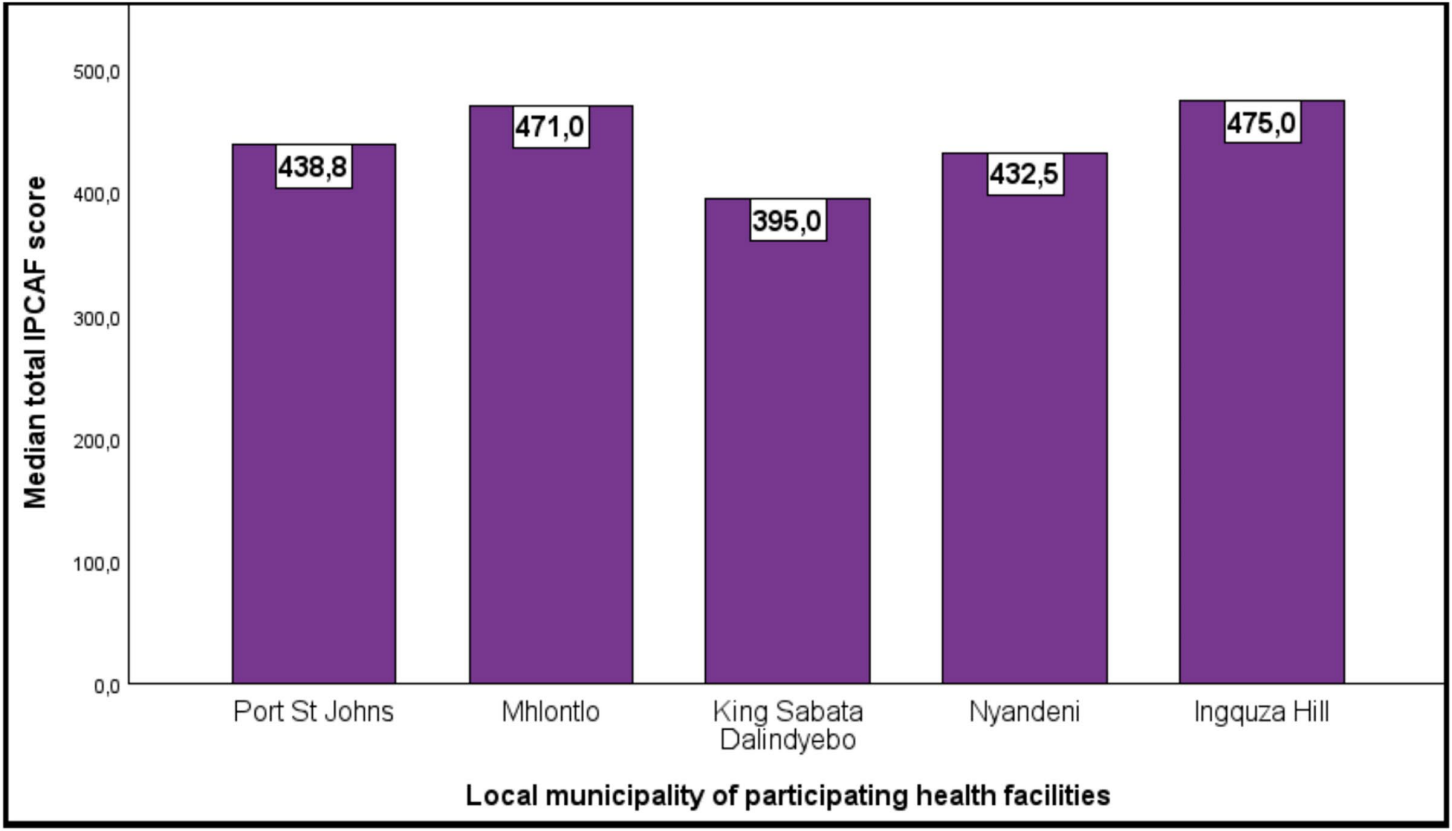
Median IPCAF scores stratified by HCF locality.

By facility location, the total IPCAF scores were highest in Ingquza Hill at 475 and lowest in KSD at 395 (Figure 4). There was no statistically significant difference in the overall median IPC assessment scores or in the median IPCAF core components across the five sub-district municipalities (Supplementary Table 1).

**Figure 5:**
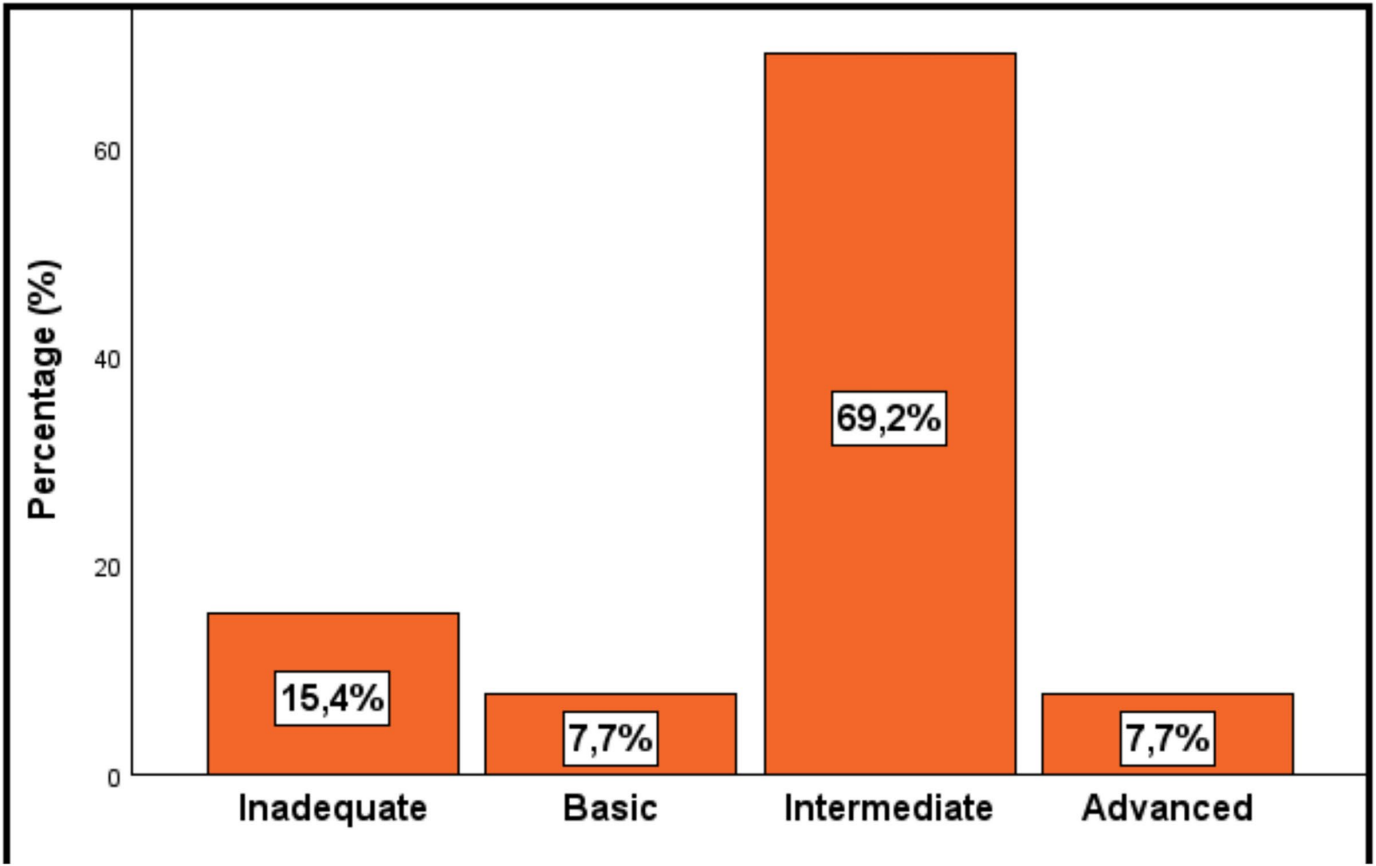
Status of IPC programs across HCFs in the O.R. Tambo district municipality.

The distribution of overall IPCAF scores across all 13 HCFs showed that 69.2% (95% CI: 42.3–88.6%, *n* = 9) of facilities were at an intermediate level, 15.4% (95% CI: 3.3–40.9%, *n* = 2) at an inadequate level, and 7.7% (95% CI: 0.8–30.7%, *n* = 1) each at the advanced and basic levels (Figure 5). There was no statistically significant difference in IPC assessment levels or in overall median IPC scores across the 13 HCFs, irrespective of facility type or location (Supplementary Table 2).

### 3.3 Analysis of IPC core component across HCFs in the OR Tambo district municipality

#### 3.3.1 Presence of an IPC program (CC1)

Of the 13 assessed HCFs, 12 (92.3%) had an IPC program in place; however, only 6 (46.2%) had clearly defined objectives and an annual activity plan, with a median score of 75.0 (interquartile range 57.5–82.5). IPC committees were present in 10 (76.9%) HCFs, and 12 (92.3%) included members of the hospital senior leadership (Table 7).

**Table 7:** Key findings of the WHO IPCAF in HCFs in the O.R. Tambo district municipality.

Core component	Expected areas	Indicators	*n* (%) *N* = 13
CC1: IPC program	IPC program availability	IPC program with clearly defined objectives and an annual activity plan	6 (46.2)
		IPC program without clearly defined objectives and an annual activity plan	6 (46.2)
		IPC team, comprising IPC professionals	9 (69.2)
		At least one full-time IPC professional or equivalent to ≤250 beds	1 (7.7)
		The IPC team or focal person has dedicated time for IPC activities	11 (84.6)
		The IPC team includes doctors and nurses	9 (69.2)
	IPC committee	The IPC committee is actively supporting the IPC team	10 (76.9)
		Senior facility leadership in IPC committee	12 (92.3)
		Senior clinical staff in IPC committee	9 (69.2)
		Facility management IPC committee	10 (76.9)
		IPC clearly defined objectives, outcome indicators, and set future targets	1 (7.7)
	Institutional support	Senior facility leadership shows clear commitment and support; the budget	2 (15.4)
		Senior facility leadership shows clear commitment and support; meeting rounds	11 (84.6)
		Microbiological laboratory support for routine day-to-day use	11 (84.6)
CC2: IPC guidelines	Available guidelines for	Facility expertise for developing or adapting guidelines	0 (0.0)
		Standard precautions	13 (100.0)
		Hand hygiene	13 (100.0)
		Transmission-based precautions	13 (100.0)
		Outbreak management and preparedness	9 (69.2)
		Prevention of surgical site infection	10 (76.9)
		Prevention of vascular catheter-associated bloodstream infections	0 (0.0)
		Prevention of hospital-acquired pneumonia	8 (61.5)
		Prevention of catheter-associated urinary tract infections	8 (61.5)
		Prevention of transmission of multidrug-resistant (MDR) pathogens	13 (100.0)
		Disinfection and sterilization	13 (100.0)
		Health care worker protection and safety	6 (46.2)
		Injection safety	13 (100.0)
		Waste management	13 (100.0)
		Antibiotic stewardship	12 (92.3)
	Guidelines development and adaptation	Guidelines are consistent with the national/international policies	13 (100.0)
		Guidelines adapted to the local context while maintaining key IPC standards	12 (92.3)
	Stakeholder involvement, training, and monitoring	Frontline healthcare workers involved in planning and executing implementation of IPC guidelines	4 (30.8)
		Relevant stakeholders involved in the development and adaptation of IPC Guidelines, in addition to IPC personnel	11 (84.6)
		Healthcare workers receive specific training on new or updated IPC guidelines introduced at the facility.	10 (76.9)
		Healthcare workers regularly monitor the implementation of at least some of the IPC guidelines in your facility	12 (92.3)
CC3: Education and training	IPC training	Personnel with IPC expertise available to lead IPC training	4 (30.8)
		Non-IPC personnel with skills to serve as trainers and mentors are available	10 (76.9)
		New employee, orientation, and regular mandatory IPC training for all HCWs at least annually	1 (7.7)
		New cleaners and other personnel involved in patient care orientation and regular IPC training are offered, but not mandatory	9 (69.2)
	
		Administrative and managerial staff receive general training regarding IPC	11 (84.6)
		Has specific IPC training for patients or family members to minimize the potential for HCAIs	1 (7.7)
	IPC training is integrated into clinical practice and other specialties	9 (69.2)
	Evaluation of IPC training programs	Has periodic evaluations of the effectiveness of training programs regularly	2 (15.4)
Ongoing IPC development for IPC staff		1 (7.7)
CC4: HAI surveillance	Organization of the surveillance system	Defined component IPC program surveillance	8 (61.5)
		Presence of personnel responsible for surveillance activities	0 (0.0)
		Personnel responsible for surveillance have been trained in epidemiology, surveillance, and IPC	2 (15.4)
		Presence of informatics/IT systems to support conducting surveillance	0 (0.0)
	Process of defining the surveillance focus areas	Prioritization exercise to determine the HAI to target surveillance	0 (0.0)
	Priority of surveillance and conducting area	Surgical site infections	5 (38.5)
		Device-associated infections	3 (23.1)
		Clinically defined infections	3 (23.1)
		Infections caused by MDR organisms	3 (23.1)
		Local priority epidemic-prone Infections	2 (15.4)
		Infections in vulnerable populations	6 (46.2)
		Infections that may affect healthcare workers	2 (15.4)
	Method of surveillance	Regular evaluation if surveillance is in line with current needs and priorities	1 (7.7)
		Use of reliable surveillance case definitions	3 (23.1)
		Use of standardized data collection methods according to international surveillance protocols	0 (0.0)
		Have processes in place to regularly review Surveillance Data	1 (7.7)
		Have adequate microbiology and laboratory to support surveillance for identifying pathogens and antimicrobial drug resistance patterns in a timely manner	8 (61.5)
	Information analysis, dissemination, and governance	Surveillance data are used to make tailored plans for the improvement of IPC practices	5 (38.5)
		Analyze antimicrobial drug resistance on a regular basis	3 (23.1)
		Provide regular feedback on up-to-date surveillance information to frontline health care workers	0 (0.0)
		Provide regular feedback on up-to-date surveillance information to \clinical leaders/heads of department	4 (30.8)
		Provide regular feedback on up-to-date surveillance information to IPC committee	4 (30.8)
		Provide regular feedback on up-to-date surveillance information to non-clinical management/administration	1 (7.7)
		Feedback on up-to-date surveillance is provided through written/oral information only	2 (15.4)
CC5: multimodal strategies for implementation of IPC interventions	Multimodal element inclusions	Use of multimodal strategies to implement IPC interventions	10 (76.9)
		System change: interventions to ensure the necessary infrastructure and continuous availability of supplies are in place	8 (61.5)
		System change: interventions to ensure the necessary infrastructure and continuous availability of supplies are in place, and addressing ergonomics and accessibility, such as the best placement of the central venous catheter set and tray	2 (15.4)
		Education and training: written information and/or oral instruction and/or e-learning only	9 (69.2)
		Monitoring and feedback: monitoring compliance with process or outcome indicators	9 (69.2)
		Communications and reminders, posters, or other advocacy/awareness-raising tools to promote the intervention	10 (76.9)
		Safety climate and culture change: managers/leaders show visible support and act as champions and role models, promoting an adaptive approach and strengthening a culture that supports IPC, patient safety and quality	1 (7.7)
	Implementation strategy	A multidisciplinary team used to implement IPC multimodal strategies	11 (84.6)
		Link colleagues from quality improvement and patient safety to develop and promote IPC multimodal strategies	11 (84.6)
		Multimodal strategies include bundles or checklists	4 (30.8)
	CC6: monitoring and audit of IPC practices and feedback	Monitoring plan	Have trained audit personnel monitoring/audit of IPC practices and feedback	8 (61.5)
			Have a well-defined monitoring plan with clear goals, targets, and activities	8 (61.5)
Monitoring indicators		Hand hygiene compliance	11 (84.6)
		Intravascular catheter insertion and/or care	5 (38.5)
		Wound dressing change	10 (76.9)
		Transmission-based precautions and isolation to prevent the spread of multidrug-resistant organisms (MDRO)	9 (69.2)
		Cleaning of the ward environment	11 (84.6)
		Disinfection and sterilization of medical equipment/instruments	11 (84.6)
		Consumption/usage of alcohol based handrub or soap	7 (53.8)
		Consumption/usage of antimicrobial agents	2 (15.4)
		Waste management	11 (84.6)
Feedback and auditing reports		Conduct the WHO hand hygiene self-assessment framework survey at least annually	12 (92.3)
		Reporting within the IPC Team	3 (23.1)
		Reporting to department leaders and managers in the areas being audited	4 (30.8)
		Reporting to frontline health care workers	0 (0.0)
		Reporting to the IPC committee or quality of care committees or equivalent	2 (15.4)
		Reporting to hospital management and senior administration	3 (23.1)
		Reporting of monitoring data undertaken regularly	9 (69.2)
		Monitoring and feedback of IPC processes and indicators performed in a “blame-free” institutional culture	9 (69.2)
		Assessment of safety and cultural factors in the facility	0 (0.0)
CC7: Workload, staffing, and bed occupancy	Staffing assessment conducted	Appropriate staffing levels assessed in your facility according to patient workload using national standards or a standard staffing needs assessment tool such as the WHO workload indicators of staffing need method	1 (7.7)
		An agreed ratio of healthcare workers to patients maintained for staff in more than 50% of units	3 (23.1)
		System in place in the facility acting on the results of the staffing needs assessments when staffing levels are deemed to be low	0 (0.0)
	Bed occupancy	The design of wards in your facility in accordance with international standards regarding bed capacity	10 (76.9)
		Bed occupancy in your facility kept to one patient per bed	11 (84.6)
		No patients are placed in beds, standing in the corridor outside of the room	3 (23.1)
		Adequate spacing of >1 m between patient beds ensured in all departments	4 (30.8)
		A system in place in your facility to assess and respond when adequate bed capacity is exceeded; this is the responsibility of the head of the department	9 (69.2)
CC8: built environment, materials and equipment for IPC at the facility level	Water	Availability of water services at all times and of sufficient quantity for all uses	7 (53.8)
		A reliable, safe drinking water station is present and accessible for staff, patients, and families at all times and in all locations/wards	7 (53.8)
	Hand hygiene and sanitation facilities	Functional hand hygiene stations with reliably available supplies at all points of care	10 (76.9)
		More than or equal to 4 toilets or improved latrines available for outpatient settings or ≥1 per 20 users for inpatient settings, sufficient number present and functional	9 (69.2)

	Power supply, ventilation, and cleaning	Sufficient energy/power supply available at day and night and in all areas for all uses	5 (38.5)
		Functional environmental ventilation available in-patient care areas	13 (100.0)
		For floors and horizontal work surfaces, is there an accessible record of cleaning, signed by the cleaners each day? Record completed and signed daily	9 (69.2)
		Appropriate and well-maintained materials for cleaning are available	7 (53.8)
	Cohering and PPE use	Single-patient rooms or rooms are available for cohorting patients with similar pathogens if the number of isolation rooms is insufficient	1 (7.7)
		A suitable room is available for patient cohorting	10 (76.9)
		PPE available at all times and in sufficient quantity for all uses for all healthcare workers	2 (15.4)
	Medical waste and sewage management	Functional waste collection containers for non-infectious (general) waste, infectious waste and, sharps waste in close proximity to all waste generation	11 (84.6)
		Functional burial pit/fenced waste dump or municipal pick-up available for disposal of non-infectious waste	7 (53.8)
		Incinerator or alternative treatment technology for the treatment of infectious and sharps waste is present but not functional	2 (15.4)
		Wastewater treatment system present and functioning reliably	8 (61.5)
	Decontamination and sterilization	Functional reliability, dedicated decontamination area, and/or sterile supply department.	10 (76.9)
		Have reliable, sterile, and disinfected equipment available every day and in sufficient quantity, ready for use	9 (69.2)
Disposable items are continuously available when necessary		10 (76.9)

#### 3.3.2 IPC guidelines available (CC2)

The IPC guidelines (CC2) had a high score of 72.5 (IQR: 70.0, 80.0), ranking among the highest-scoring core components. Guidelines for standard precautions, hand hygiene, transmission-based precautions, prevention of transmission of multidrug-resistant (MDR) pathogens, disinfection and sterilization, injection safety, and waste management were the most available, present in all assessed HCFs (100%). By contrast, guidelines for preventing vascular catheter-associated bloodstream infections were the least available, with none of the HCFs having them. More than half of the assessed HCFs had guidelines for preventing surgical site infections (76.9%), hospital-acquired pneumonia (61.5%), and catheter-associated urinary tract infections (61.5%). Antibiotic stewardship guidelines were available in 12 (92.3%) HCFs.

Overall, all HCFs (100%) had IPC guidelines consistent with national and/or international policies, and 11 (84.6%) involved relevant stakeholders in developing these guidelines. In 10 (76.9%) HCFs, healthcare workers had been trained on new or updated IPC guidelines, whereas 12 (92.3%) facilities reported monitoring healthcare workers’ adherence to these guidelines (Table 7).

#### 3.3.3 IPC education and training (CC3)

The median score for this core component was 45.0 (IQR: 45.0, 60.0). In 4 (30.8%) HCFs, IPC training was delivered by personnel with formal IPC expertise. However, in almost all HCFs, mandatory annual IPC training for all healthcare workers was not consistently implemented, with only 1 (7.7%) hospital reporting this practice. Administrative and managerial staff from 11 (84.6%) HCFs underwent IPC training, whereas only 1 (7.7%) study site provided IPC education to patients and their family members. Only 2 (15.4%) HCFs regularly evaluated their IPC training programs (Table 7).

#### 3.3.4 HAI surveillance activities (CC4)

The lowest median score was observed in HAI surveillance, at 20.0 (IQR: 0.0, 32.5). Surveillance programs were implemented in 8 (61.5%) HCFs, none of which used informatics or technology systems to support surveillance. Only 2 (15.4%) HCFs had personnel with basic epidemiology and surveillance training responsible for surveillance activities. Infections caused by multidrug-resistant (MDR) pathogens and device-associated infections were less frequently surveilled, each at 3 HCFs (23.1%), whereas surgical site infections were most frequently surveilled at 5 HCFs (38.5%). Regular surveillance evaluations occurred in only 1 (7.7%) hospital, and only 3 (23.1%) HCFs used standardized surveillance case definitions. Microbiology-based surveillance was conducted in a limited number of HCFs, with laboratory capacity in 8 (61.5%) facilities (Table 7).

#### 3.3.5 Multimodal strategies (CC5)

In 10 (76.9%) HCFs, a multimodal IPC implementation strategy was used, with a median score of 60.0 (IQR: 60.0, 65.0). System-level interventions to ensure infrastructure and the continuous availability of supplies were reported in 8 (61.5%) HCFs. Additionally, only 1 (7.7%) hospital reported that managers and leaders actively supported and served as role models for safety and culture change initiatives (Table 7).

#### 3.3.6 Monitoring and audit of IPC practices and feedback (CC6)

Core component six (CC6) had a relatively high median score of 67.5 (IQR: 47.5, 75.0). Trained staff to monitor and audit IPC activities were available in 8 (61.5%) HCFs, and a similar proportion (61.5%) had a well-defined monitoring plan with clear objectives and targets. Certain indicators were monitored more frequently than others: hand hygiene (84.6%), waste management (84.6%), disinfection and sterilization, and cleaning (each at 84.6%). Other indicators, including antimicrobial consumption, were monitored in fewer than a third of the assessed HCFs. The WHO hand hygiene self-assessment framework was conducted regularly in 12 (92.3%) HCFs (Table 7).

#### 3.3.7 Workload, staffing, and bed occupancy (CC7)

The median workload, staffing, and bed occupancy score was 30.0 (IQR: 20.0, 30.0). Staffing levels aligned with patient workload were assessed in only 1 (7.7%) hospital. The patient-to-healthcare worker ratio was maintained in 3 HCFs (23.1%). Bed arrangements based on international standards were present in 10 (76.9%) HCFs. Adequate bed spacing of more than 1 m was ensured in only 4 HCFs (30.8%) (Table 7).

#### 3.3.8 Environment, materials, and equipment for IPC (CC8)

The median scores for environment, materials, and equipment were 72.5 (IQR: 62.5, 77.5). Sufficient water supply and access to safe drinking water were available in 7 (53.8%) HCFs for general use. Functional hand hygiene stations and adequate toilet facilities were present in 10 (76.9%) and 9 (69.2%) HCFs, respectively. A sufficient and continuous power supply was available in 5 (38.5%) HCFs, whereas functional environmental ventilation was present in all HCFs (100%). Continuous availability of personal protective equipment (PPE) for healthcare workers was reported in only 2 (15.4%) HCFs, and functional waste segregation containers for different waste types were present in the majority of HCFs (84.6%) (Table 7).

## 4 Discussion

The assessment of IPC practices in the Eastern Cape found that most evaluated HCFs were at an intermediate level (69.2%), while only one facility achieved advanced status (7.7%). An intermediate level of IPC typically indicates that basic structures for sustainable practices are in place, but challenges remain in achieving consistent, long-term sustainability (7, 16). This distribution shows that IPC practices were adopted across most sites, but efforts must now focus on uplifting those at inadequate and basic levels and on supporting intermediate sites as they transition toward advanced IPC performance. Sustained investment in training, monitoring, and resource allocation will be critical to strengthening the health system’s ability to prevent and control infections. Similar to our study, Ng’ambi et al. (15) reported that 66.7% of facilities were at the intermediate level, 26.4% at the basic level, and 6.9% at the advanced level. Our findings on IPC capacity at basic and inadequate levels (≈23%) indicate that patients and staff in these facilities are at heightened risk of HAIs and AMR transmission, and that IPC capacity is inequitable across the service platform. This is a concerning situation that exposes systemic barriers, including inadequate staffing, weak surveillance systems, and insufficient training. In a cross-sectional study of 65 HCFs in Cameroon, 86% of facilities were inadequate or basic, and none were advanced (17). Furthermore, the first WHO global IPCAF survey (81 countries; 4,440 facilities) found that many facilities worldwide were at basic or inadequate levels, particularly in low-income countries, whereas high-income settings had much higher proportions of advanced facilities (2).

Global evidence shows that the highest IPC CC scores in HCFs were in CC8 (built environment) and CC2 (IPC guidelines) (2), whereas the lowest scores were in CC7 (workload, staffing, and bed occupancy), CC5 (multimodal strategies), and CC3 (IPC education and training) (2, 18, 19). In LMICs, CC4 (HAI surveillance) and CC6 (monitoring IPC implementation and feedback) were the lowest-scoring CCs (2, 17). In our study, the highest-scoring components were CC1, followed by CC2 and CC8, while the lowest-scoring components were CC4, CC7, and CC3. Our findings are similar to those reported in DRC and Zambia, where CC1 and CC2 were highest (16, 20). This aligns with established global guidelines for establishing IPC and developing IPC resources for LMICs (1, 21). In contrast, reports from Burkina Faso documented that the highest IPCAF scores were in CC4 and CC8 (20). Strong performance in IPC guidelines and programs suggests that HCFs generally have adequate workplace policies and reasonable adherence (22). Strong infrastructure scores indicate the availability of materials and physical resources on most sites. However, IPC capacity in the Eastern Cape is constrained by management issues, resource limitations, inadequate training, poor adherence to protocols, and a lack of local benchmarks for comparison (23). Poor IPC capacity leads to increased HAIs and AMR (22, 24).

Furthermore, the present study found an IPCAF score of 452.5. These findings are similar to those reported across 33 hospitals in Malawi, with an IPCAF score of 445 (22), and in Burkina Faso, with an IPCAF overall score of 415 (20), suggesting more significant challenges with basic IPC infrastructure and implementation in those contexts. Among hospitals with intermediate IPC capacity, higher IPCAF scores were reported in nine hospitals in Zambia (594; 16), six in Uganda (547; 34), and 25 in Rwanda (545; 14). Other studies from the Democratic Republic of Congo (DRC) and Côte d’Ivoire reported basic IPC capacity, with IPCAF scores of 392.5 and 242.5, respectively (20, 25). Many LMICs have reported key barriers, including insufficient IPC budget allocation, unclear IPC aims and objectives, erratic supplies of IPC materials, issues with staff training, inadequate staffing with full-time IPC experts, and a lack of HAI surveillance and periodic monitoring (3, 26). In contrast to our findings, data from the first WHO global survey on IPC in HCFs across 81 countries, as well as from Austria, Turkey, China, Germany, Japan, and Latin America, reported advanced IPC capacity (2, 19, 23, 27–30), with IPCAF median scores ranging from 605 to 692.5. This may reflect well-established IPC practices, adequate IPC staffing, high-income levels in these countries, and increased funding for healthcare infrastructure (16). Taken together, these findings reveal a disparity in IPC capacity between LMICs and high-income countries (HIC), with lower IPC scores observed in LMICs and in public HCFs (31).

HAI surveillance in this study had the lowest median IPCAF score, indicating it is one of the weakest IPC core components across the assessed facilities, consistent with findings from other LMICs, such as Malawi and India. These studies also report that HAI surveillance is poorly developed, even when surveillance activities are in place (15, 32). In this study, the low score reflects both gaps in availability and limitations in quality. Although 61.5% of facilities reported conducting surveillance, few used standardized case definitions, only one regularly evaluated surveillance activities, and none used informatics systems. This suggests that surveillance was largely informal and not fully aligned with WHO recommendations for systematic, data-driven monitoring (33).

Human resource constraints further undermine surveillance capacity. Only 15.4% of facilities had staff trained in basic epidemiology or surveillance, a figure that mirrors challenges reported in South African public hospitals (24). These constraints are likely more severe in primary- and secondary-level facilities, where IPC responsibilities are often added to routine clinical duties. Structural factors, including limited governance, financing, and electronic data systems, also contribute to weak surveillance performance. The absence of informatics-supported surveillance across all facilities underscores reliance on manual systems, which limit timely analysis and feedback. Surveillance for multidrug-resistant organisms and device-associated infections was limited, likely reflecting restricted microbiology and diagnostic capacity.

The low median IPCAF scores for workload, staffing, and bed occupancy in the present study indicate substantial limitations in human resource capacity and patient accommodation practices within the assessed healthcare facilities. Similar concerns regarding staffing shortages and overcrowding have been reported in studies conducted in LMICs using the WHO IPCAF tool. Low IPCAF core component scores for workload, staffing, and bed occupancy have also been reported in other LMICs, including Malawi, India, Rwanda, and Zambia (14–16, 32). However, comparisons with these studies should be interpreted cautiously due to significant methodological and contextual differences. These studies were conducted in tertiary and specialized referral hospitals and in secondary-level HCFs with more complex patient profiles and larger bed capacities, whereas the present study included primary-level HCFs in a rural district municipality. Differences in healthcare system organization, patient volumes, infrastructure, and availability of specialized IPC personnel may therefore influence IPC scores and limit direct comparability across settings.

The low proportion of facilities assessing staffing levels relative to workload may reflect limited workforce planning and monitoring systems in the district healthcare context. In many resource-constrained public healthcare systems, staffing allocation is often influenced by broader operational pressures, workforce shortages, and budgetary constraints rather than by formal workload assessment models. Rural healthcare facilities may face additional challenges in recruiting and retaining healthcare workers, further affecting staffing adequacy and the continuity of IPC activities. Similar observations have been reported in South African public HCFs, where shortages of trained personnel, increased workload, and limited institutional support have affected IPC capacity (24).

### 4.1 Implications for policy and practice

There is a need for targeted support for basic and inadequate facilities. Facilities performing at basic or inadequate levels should be prioritized for IPC resources, staff training, mentorship, and infrastructural strengthening, as they present the highest safety risks. Scaling up strong-performing facilities as IPC hubs is encouraged to serve as mentorship and quality-improvement hubs, facilitating peer learning, supervision, and SOP harmonization. Standardized processes for HAI surveillance and regular data review should be developed, and dedicated IPC focal persons, structured IPC committees, and sustainable budget lines for PPE, disinfectants, hand hygiene commodities, and training should be mandatory within district and facility budgets. IPCAF indicators should be integrated into routine quality and AMR systems to enable routine performance monitoring, accountability, and targeted supervision. Periodic IPCAF reassessment would allow facilities to document progress, address persistent gaps, and evaluate the impact of system-strengthening interventions.

Overall, the study contributes context-specific evidence that may support ongoing efforts to strengthen IPC programs and improve healthcare quality within the Eastern Cape Province.

### 4.2 Limitations

This study is not without limitations. First, the analysis was conducted in only 13 facilities, limiting generalizability to all health facilities in the region, including regional, tertiary, and private facilities, because IPC capacity and organizational structures may differ across HCF levels. The non-participation of some eligible facilities may have affected the representativeness of the findings, so the findings may not fully reflect IPC capacity across all HCF categories in the district. Second, the cross-sectional design precludes trend analysis or measurement of improvement over time. Third, IPCAF scores are self-reported, introducing potential reporting bias and variation in scoring accuracy. The facility representative may have over-reported compliance with IPC practices or facility preparedness due to social desirability bias or perceived performance expectations. Fourth, the study did not triangulate IPCAF performance with microbiological data, real-time audit findings, or actual HAI incidence, which would have enabled stronger correlations between IPC maturity and patient outcomes. A further limitation is that the limited number of participating facilities may have limited the ability to detect statistically significant differences between facility categories or IPC domains. Also, multiple comparisons were conducted without adjustment, increasing the risk of a Type I error. Given the exploratory nature and small sample size, the findings should be interpreted cautiously. Despite these limitations, the findings provide valuable preliminary evidence that warrants further investigation in larger, confirmatory studies.

## 5 Conclusion

The assessed healthcare facilities demonstrated predominantly intermediate IPC capacity, with notable gaps in HAIs surveillance, IPC education and training, workload and staffing, and monitoring systems. These findings underscore the need to strengthen key IPC core components within the HCFs to support more consistent IPC capacity across the district. They also contribute to ongoing efforts to strengthen and optimize IPC programs in the Eastern Cape by supporting alignment with global IPC standards and addressing contextual healthcare challenges in resource-limited settings.

### 5.1 Recommendations

Priority areas for strengthening IPC capacity in the assessed HCFs include standardizing HAI surveillance systems by improving documentation, adopting standardized case definitions, conducting regular evaluations, and implementing timely feedback mechanisms. The absence of informatics-supported surveillance indicates a need to gradually integrate simple electronic surveillance and reporting systems into existing healthcare structures.

Regular in-service IPC training and refresher programs should be strengthened to improve adherence to IPC practices. HCFs should also strengthen monitoring and auditing by establishing functional IPC committees, providing regular supervisory feedback, and clearly defining IPC objectives and action plans. The study further recommends that facility managers improve workforce planning, patient flow management, and bed spacing, where feasible. Further studies involving larger and more diverse HCFs are warranted to provide broader evidence on IPC capacity in the Eastern Cape Province.

In the long term, district and provincial health authorities in the Eastern Cape should prioritize sustained investment in IPC infrastructure, digital health systems, workforce development, and dedicated support for IPC programs to strengthen IPC capacity across HCFs.

## Data Availability

The original contributions presented in the study are included in the article/Supplementary material, further inquiries can be directed to the corresponding authors.
