# Supplementary material for: Assessment of infection prevention and control practices in healthcare facilities in the O.R. Tambo district municipality using the WHO infection prevention and control assessment framework
Source: Front Public Health. 2026 Jun 5;14:1772386. doi: 10.3389/fpubh.2026.1772386 (PMC13279705; doi:10.3389/fpubh.2026.1772386)
Supplement: Supplementary file 1 [file Supplementary_file_1.docx]

Supplementary Table 1. Comparison of IPCAF total scores across participating health facilities within local municipalities in the O.R Tambo district municipality

| **Core component** | **Location of facility; Median (Interquartile range)** | | | | | **p-value** |
| --- | --- | --- | --- | --- | --- | --- |
|  | **Port St Johns, N=** | **Mhlontlo** | **King Sabata Dalindyebo** | **Nyandeni** | **Ingquza Hill** |  |
| **CC1** | 73.8 (65.0–82.5) | 77.5 (57.5–82.5) | 65.0 (43.8–80.0) | 75.0 (75.0–75.0) | 70.0 (70.0–70.0) | 0.975 |
| **CC2** | 75.0 (70.0–80.0) | 80.0 (77.5–80.0) | 67.5 (62.5–70.0) | 92.5 (92.5–92.5) | 72.5 (72.5–72.5) | 0.130 |
| **CC3** | 45.0 (45.0–45.0) | 45.0 (45.0–60.0) | 52.5 (25.0–60.0) | 40.0 (40.0–40.0) | 60.0 (60.0–60.0) | 0.632 |
| **CC4** | 25.0 (20.0–30.0) | 40.0 (20.0–52.5) | 8.8 (0.0–25.0) | 0.0 (0.0–0.0) | 17.5 (17.5–17.5) | 0.360 |
| **CC5** | 62.5 (60.0–65.0) | 60.0 (60.0–65.0) | 47.5 (17.5–60.0) | 65.0 (65.0–65.0) | 70.0 (70.0–70.0) | 0.259 |
| **CC6** | 71.3 (67.5–75.0) | 47.5 (42.5–52.5) | 71.3 (53.8–75.0) | 57.5 (57.5–57.5) | 75.0 (75.0–75.0) | 0.433 |
| **CC7** | 25.0 (20.0–30.0) | 45.0 (30.0–55.0) | 17.5 (10.0–27.5) | 30.0 (30.0–30.0) | 25.0 (25.0–25.0) | 0.514 |
| **CC8** | 61.3 (60.0–62.5) | 77.5 (72.5–78.5) | 71.3 (42.5–73.8) | 72.5 (72.5–72.5) | 85.0 (85.0–85.0) | 0.258 |
| Total IPCAF score | 438.8 (425.0–452.5) | 471.0 (467.0–501.0) | 395.0 (268.8–457.5) | 432.5 (432.5–432.5) | 475.0 (475.0–475.0) | 0.374 |

Supplementary Table 2. Distribution of IPCAF levels by facility type and local municipality

| Variable of interest | IPCAF categories | | | | p-value |
| --- | --- | --- | --- | --- | --- |
|  | Inadequate n (%) | Basic n (%) | Intermediate n (%) | Advanced n (%) |  |
| Facility type |  |  |  |  |  |
| Community Health Centres | 2 (100.0) | 0 (0.0) | 6 (66.7) | 0 (0.0) | 0.249* |
| District Hospitals | 0 (0.0) | 1 (100.0) | 3 (33.3) | 1 (100.0) |  |
| Local municipality of participating health facilities |  |  |  |  |  |
| Port St Johns | 0 (0.0) | 0 (0.0) | 2 (22.2) | 0 (0.0) | 1.000* |
| Mhlontlo | 1 (50.0) | 0 (0.0) | 3 (33.3) | 1 (100.0) |  |
| King Sabata Dalindyebo | 1 (50.0) | 1 (100.0) | 2 (22.2) | 0 (0.0) |  |
| Nyandeni | 0 (0.0) | 0 (0.0) | 1 (11.1) | 0 (0.0) |  |
| Ingquza Hill | 0 (0.0) | 0 (0.0) | 1 (11.1) | 0 (0.0) |  |

**Fisher–Freeman–Halton exact test (Monte Carlo, two‑sided)*
